# Viral exposure and its effects on subsequent development of a mental illness: Results from the VIRAL-MI consortium project

**DOI:** 10.1192/j.eurpsy.2025.59

**Published:** 2025-08-26

**Authors:** D. Tzur Bitan, VIRAL-MI consortium

**Affiliations:** Community Mental Health, University of Haifa, Haifa, Israel

## Abstract

**Abstract: Background:**

Infectious diseases are known to significantly increase the risk for later development of a mental disorder. Nonetheless, only few studies utilized big-data strategies to evaluate whether cumulative viral infection increases the risk of a subsequent mental illness, and whether risk and resilience factors a likely to modify theses associations. Methods: This study assessed the likelihood of mental illness development among individuals with recurrent exposure to pre-determined viruses (n = 53,416). The viral blood tests included in the analysis was based on consensus agreement of the VIRAL-MI consortium members, and was based on test validity, mandatory vaccination profiles, and the test’s ability to detect acute infection. Broadly, these tests included positivity to the following viral infections: Cytomegalovirus, Epstein-Barr virus, Hepatitis, Herpes, Toxoplasma and Influenza. Data was obtained through the Clalit Health Services, the largest healthcare organization in Israel. Results: Overall, 663,231 tests were obtained from the cohort of 53,416 cases. Kaplan-Meier analyses indicated that individuals infected with more than three viruses were significantly more likely to develop a mental disorder (Log rank 14.55, p<.001). Of the investigated disorders, only anxiety disorders presented a significantly differential pattern of cumulative risk, in the same direction (Log rank 10.06, p = .007). Conclusions: Cumulative viral infections may have significant contribution to the development of a mental disorder. Limitations and integration with previous research will be discussed.

**Disclosure of Interest:**

None Declared

